# A longitudinal study of theory of mind across the lifespan

**DOI:** 10.3389/fpsyg.2025.1549378

**Published:** 2025-07-17

**Authors:** Hanna G. Erceg, Ruby S. Dhillon, Daniel G. Derksen, Eric Y. Mah, Daniel M. Bernstein

**Affiliations:** ^1^Department of Psychology, Kwantlen Polytechnic University, Surrey, BC, Canada; ^2^Department of Psychology, Simon Fraser University, Burnaby, BC, Canada; ^3^Department of Psychology, University of Victoria, Victoria, BC, Canada

**Keywords:** theory of mind, longitudinal analysis, lifespan, Sandbox task, reading the mind in the eyes, social cognition, cognitive and affective ToM, false-belief understanding

## Abstract

**Introduction:**

Theory of Mind (ToM) is essential for social interactions. However, gaps remain in our knowledge of when ToM abilities develop and change, particularly from adolescence to older adulthood.

**Methods:**

We used data from an ongoing longitudinal study to examine ToM abilities across three time points in participants aged 3 years and older. Testing waves occurred over multiple years. Cognitive ToM was assessed using the Sandbox task (*N* = 187; age range = 3–80 years), and affective ToM was assessed using the Reading the Mind in the Eyes Task (RMET; *N* = 121; age range = 6–80 years). Data were analyzed using mixed-design ANOVAs to examine interactions between Age Group and Time Point.

**Results:**

Children aged 6–9 years exhibited significantly lower ToM abilities compared to adults. However, beyond childhood, both cognitive and affective ToM remained relatively stable across the lifespan.

**Discussion:**

Our study illuminates critical periods of ToM development. Moreover, our study highlights the importance of using measures that capture subtle changes across the lifespan.

## Introduction

### A longitudinal study of theory of mind across the lifespan

Theory of Mind (ToM) is the ability to attribute mental states to oneself and others. Specifically, ToM is the ability to understand and reason about beliefs, desires, thoughts, intentions, and feelings (Premack and Woodruff, 1978; Wimmer and Perner, 1983). ToM plays a crucial role in everyday social interactions. However, despite over four decades of research, gaps remain in our knowledge of when ToM abilities develop and change, particularly across the adolescent to older adult lifespan (Derksen et al., 2018). While developmental patterns of ToM have been explored, much of our knowledge comes from cross-sectional studies. There is, however, longitudinal research focusing on children (see Wellman, 2014). Our study utilized a longitudinal design to explore ToM abilities across the child to older adult lifespan to provide a more comprehensive understanding of when these abilities develop and change. Our results highlight critical periods of ToM development.

### Developmental patterns

Research has explored the developmental patterns of ToM abilities. Cross-sectional studies suggest that ToM abilities improve from preschool age to adolescence, stabilize from adolescence through adulthood, and then decline in older adulthood (Cornaggia et al., 2024; Dumontheil et al., 2010; Henry et al., 2013; Kong et al., 2025; Miller, 2022; Tousignant et al., 2017; Wellman et al., 2001). Notably, both cross-sectional and longitudinal research has primarily focused on children aged 3-13. These studies suggest that ToM mastery follows a predictable development of related skills in the following order: (a) diverse desires (understanding that different people can want different things), (b) diverse beliefs (understanding that opinions can differ), (c) knowledge access (not seeing = ignorance), (d) false belief understanding, (e) hidden emotion (people can conceal their true feelings behind false facial expressions), and (f) sarcasm (Peterson and Wellman, 2019; Wellman et al., 2011; Wellman and Liu, 2004)^1^. Research beyond middle childhood, especially longitudinal studies, is relatively sparse (see Derksen et al., 2018). As a result, there is a heavy reliance on cross-sectional designs, which limits the conclusions that can be drawn from the existing literature.

It remains unclear whether the observed developmental differences across the adolescent to older adult lifespan reflect true age-related changes or are merely the result of different task demands across the various measures used to assess ToM in different age groups. It is also possible that the observed differences reflect the development of the various skills necessary to complete ToM tasks (e.g., executive function, working memory) that vary across ToM tasks of different complexities. To address these concerns, longitudinal research using a single task to assess ToM across age groups is needed. Notably, discrete measures of ToM, such as the categorical change-in-location task known as the Sally-Anne task (Wimmer and Perner, 1983), are more common than continuous measures (e.g., reaction time, eye-tracking, mouse trajectory; Apperly et al., 2011; Keysar et al., 2003; O'Connor et al., 2024). Discrete measures may oversimplify the developmental trajectory of ToM, potentially exaggerating differences between age groups. For instance, a pass/fail coding system could make developmental changes appear more pronounced by masking subtle, continuous development. Thus, developmental patterns in ToM might be more subtle than previously believed.

To address concerns about the use of different ToM tasks across age groups and the limitations of discrete measures in capturing subtle developmental changes, Sommerville et al. (2013) developed the Sandbox task to measure ToM as a continuous (rather than categorical) variable. The Sandbox task is a modified change-of-location task appropriate for measuring false-belief understanding in preschoolers through older adults and also great apes (Lurz et al., 2022; c.f., Haskaraca et al., 2023; Samuel et al., 2018). Research using the Sandbox task reveals differing developmental patterns from those found using discrete measures in different age groups. While the existing literature shows striking developmental differences in ToM abilities across the child to older adult lifespan, cross-sectional work using the Sandbox task reveals that ToM abilities remain relatively stable from preschool to older age (Bernstein, 2021).

### Components of theory of mind

Utilizing a single measure across age groups can address some limitations of past research, which has used various measures to assess ToM. However, understanding the developmental patterns of the distinct components of ToM is equally important. Notably, ToM consists of two main components: cognitive and affective (Shamay-Tsoory and Aharon-Peretz, 2007; see also Meinhardt-Injac et al., 2020). Cognitive ToM refers to the ability to understand the beliefs, intentions, and desires of oneself and others, while affective ToM refers to the ability to recognize and understand the emotions and feelings of others. Fewer studies have explored affective ToM than cognitive ToM (Mahy, 2018).

One way to measure affective ToM is with the Reading the Mind in the Eyes Task (RMET; Baron-Cohen et al., 2001). In the RMET, participants view pictures of eyes and indicate the matching emotion from a list. The RMET presents pictures of people's complex emotions in social situations. This is considered an advanced ToM ability (c.f., Higgins et al., 2024; Oakley et al., 2016) because a relevant social context must be referenced from memory to understand the emotion (Baron-Cohen et al., 2001, 1997a,b).

Affective ToM tends to decline earlier than cognitive ToM in older adults, highlighting a need for research to distinguish between the two components (Raimo et al., 2022; c.f., Bottiroli et al., 2016). One possible explanation for affective ToM declining earlier might relate to social interaction. Social interaction influences the development of ToM abilities. Increased social interactions enhance these abilities, presumably by providing more opportunities to practice inferring others' mental states (Yu and Wellman, 2023). Therefore, it is plausible that the observed decline in affective ToM around age 60 is related to the decrease in social interactions older adults experience during this life stage. However, a more likely explanation might be that declines in affective ToM are due to age-related declines in episodic memory, which is a specific cognitive ability. As noted earlier, the RMET requires participants to match expressions around the eyes to stored examples of relevant context from past experiences (Baron-Cohen et al., 2001). As episodic memory declines with age (Levine et al., 2002; Rönnlund et al., 2005), older adults may struggle to retrieve these episodes, leading to poorer affective ToM. Alternatively, cognitive ToM may decline later because of age-related declines in more general cognitive abilities. This understanding is supported by work revealing that executive function mediates age-related declines in cognitive ToM (Charlton et al., 2009; Phillips et al., 2011). Moreover, some work suggests that such age-related declines in cognitive ToM are due to age-related changes in executive functioning and not merely to declines in ToM competence (Cho and Cohen, 2019). However, this view is debated, and other work suggests cognitive ToM declines due to factors beyond task demands or general cognitive abilities, reflecting a decline in ToM competence itself (Bernstein et al., 2011; Bloom and German, 2000). Overall, the literature to date suggests future work would benefit from measuring both cognitive and affective ToM abilities as distinct constructs.

### The present study

To expand the existing literature on ToM, there is a need for longitudinal research that uses a single measure of ToM across age groups and distinguishes between cognitive and affective components. While longitudinal research on ToM exists, it has largely focused on preschool-aged children and adolescents (see Derksen et al., 2018). To our knowledge, there is currently no longitudinal work on ToM in adults.

This study seeks to advance our understanding of the developmental trajectory of ToM across the lifespan. We conducted a longitudinal analysis to explore age-related changes in ToM ability. We included separate measures of cognitive and affective ToM and used the same tasks across different age groups, spanning preschool to older adulthood. Based on previous literature, we hypothesized that: (1) Cognitive ToM would remain relatively stable from preschool to adulthood, with modest declines in older adulthood; (2) Affective ToM would remain relatively stable from childhood to adulthood, with modest declines in older adulthood emerging earlier compared to cognitive ToM.

## Materials and methods

This research was conducted using data collected from an ongoing longitudinal study at a mid-sized University in Western Canada. Recruitment for the study started in 2015. Participants were recruited through various strategies. Children were recruited through local schools and community events, and older adults were recruited through community centers and independent living facilities. Undergraduate students were primarily recruited from the university's subject pool.

Participants completed a battery of measures, including the Sandbox task (Sommerville et al., 2013) and the RMET (Olderbak et al., 2015). To allow for within-subject comparisons over time, we limited our analyses to participants who had completed three waves of testing for each task. This decision preserved the integrity of the mixed-design ANOVAs, which require repeated measures across all included time points. For participants with more than three waves of data, we included their first three waves in the analysis.

A total of 696 participants completed at least one wave of testing for the Sandbox task, and 588 participants completed at least one wave of testing for the RMET. However, only a subset of participants returned and completed additional waves of testing for each task. As a result, a total of 187 participants completed three waves of testing for the Sandbox task (65.2% female, 34.8% male; mean age = 28.4 years, *SD* = 24.9, range = 3.06–80.2 years) and 121 participants completed three waves of testing for the RMET (67.8% female, 32.2% male; mean age = 31.1 years, *SD* = 24.3, range = 6.1–80.2 years). The average delay between time points for the Sandbox sample was 2.17 years (*SD* = 1.73), and 2.3 years (*SD* = 1.67) for the RMET sample.

Participants were grouped into age categories to reflect developmental stages (see Bernstein, 2021). Age groups for the Sandbox task included: 3–5 years (*N* = 31), 6–9 years (*N* = 40), 10–17 years (*N* = 32), 18–64 years (*N* = 58), 65+ years (*N* = 26). Age groups for the RMET included: 6–9 years (*N* = 36), 10–17 years (*N* = 25), 18–64 years (*N* = 45), 65+ years (*N* = 15). There were some differences in the average delay between time points across age groups (e.g., 65+ years had longer delays between waves than some other age groups, and the delay between Time Points 2–3 was on average longer than the delay between Time Points 1–2; see Supplementary material 1b and 2b for more details).

### Measures

#### Sandbox task

The Sandbox Task is a modified change-of-location task used to measure cognitive ToM. Specifically, the Sandbox Task measures false-belief understanding as a continuous variable (Sommerville et al., 2013). Participants hear four short stories. In each story, an experimenter buries an object in a large box filled with Styrofoam at an initial location in the protagonist's view (L1). The protagonist then leaves. While the protagonist is absent, a second character digs up the item and moves it to a new location (L2), once again burying the item so that it is out of view from the protagonist. Participants then complete a 20-s visual search filler task before answering a false-belief and/or a memory-control question. In false belief questions, participants indicate where in the sandbox the protagonist would look for the item upon returning (requiring them to adopt the protagonist's perspective). In memory-control questions, participants are asked to recall where the item was initially placed (L1). In both cases participants respond by pointing to a location in the Sandbox. Experimenters record the response using a tape measure along the Sandbox's inside seam (visible only to the experimenter). We administered the task to participants 3 years and older. Prior to 2018, participants answered only one test question at the end of each trial: either a false-belief question or a memory-control question. Starting in 2018, participants answered both a false-belief and memory-control question for each story, doubling the amount of data collected. The majority (63%) of testing instances were collected using the single-question version of the task.

**Figure 1:**
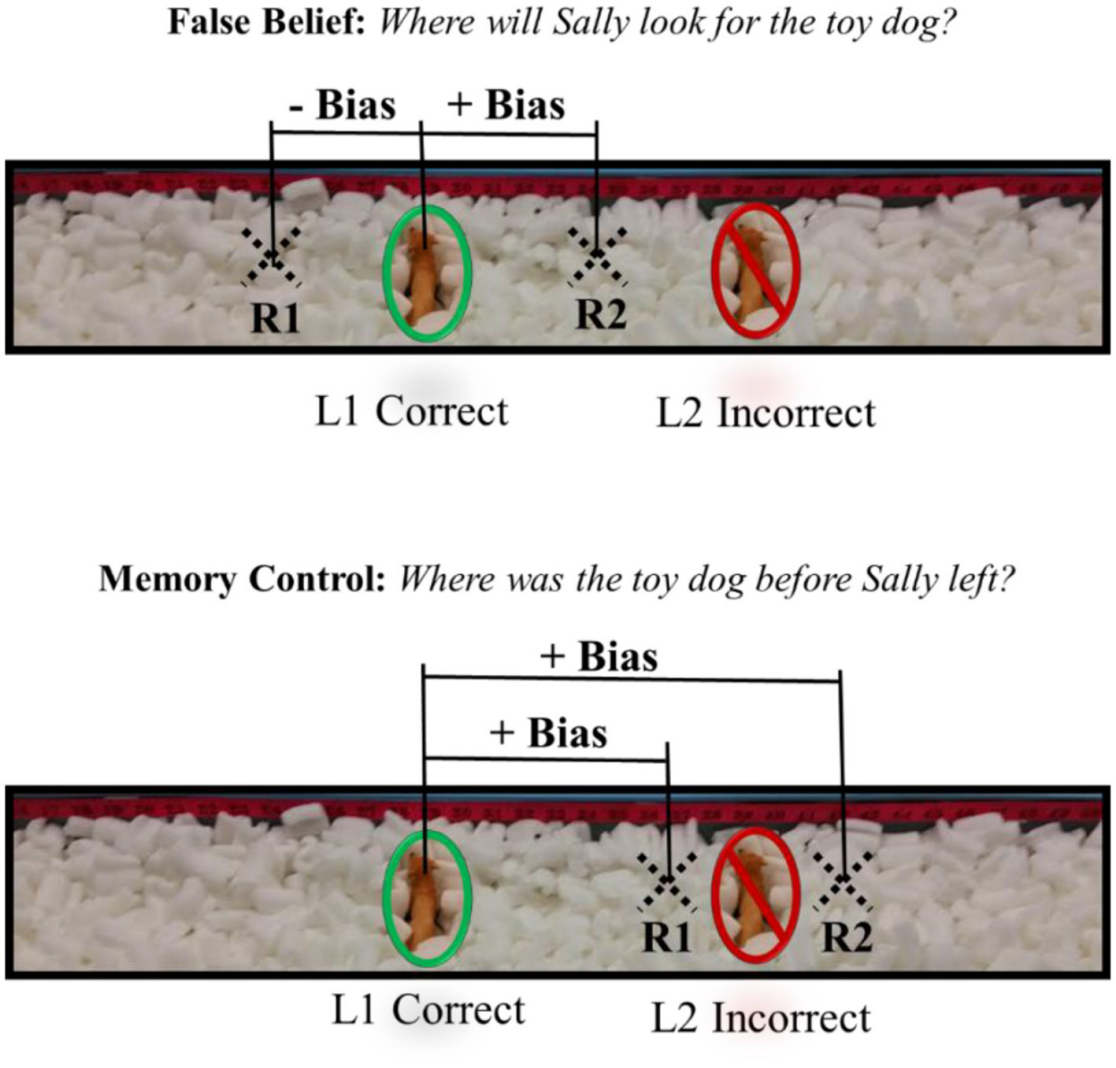
Bias calculation in the Sandbox task. L1 refers to the original location of the hidden object. L2 refers to the new location of the object after it was moved. R1 and R2 are examples of possible responses (i.e., Response 1 and Response 2). In all cases the object was not visible to the participant during their responses to test questions. In the False Belief example, a response at R1 would produce a negative bias because the response moves away from the incorrect location. A response at R2 would produce a positive bias because the response moves toward the incorrect location. In the Memory Control example, both responses would produce positive bias, however, a response at R2 would produce a larger positive bias as the response is even further from the correct location.

Bias scores were calculated separately for false-belief and memory-control trials. For each test question, responses toward the incorrect location denoted positive bias, and responses away from the incorrect location denoted negative bias (see Figure 1). An egocentric bias score was then calculated by subtracting the memory-control bias score from the false-belief bias score. Higher egocentric bias scores indicate greater difficulty suppressing one's own knowledge of the true location (L1) when reasoning about others' perspectives. Thus, a higher egocentric bias score reflects poorer false-belief reasoning, a key aspect of cognitive ToM.

#### Reading the mind in the eyes task (RMET)

We administered the RMET to assess affective ToM in participants ages 6 years and older (Baron-Cohen et al., 2001). For adults, we initially used a shortened 12-item version of the RMET, but starting in 2019, we adjusted this to a 10-item version, which demonstrated better reliability compared to the original 36-item version (Olderbak et al., 2015). In our analysis sample (restricted to the first three completed waves of data for participants who had at least three waves of data), only one testing instance used the 10-item version. Children aged 6 to 17 years consistently received a 12-item version adapted with age-appropriate vocabulary. Preschoolers did not complete the RMET. Participants viewed a series of grayscale photographs depicting only the eye regions of various individuals. After each photograph appeared, participants tried to identify the emotional state that best represented the individual by choosing among four options. To ensure comprehension, participants received a list of definitions for each emotional state option. There was no time limit on the task. To account for the use of different task versions (i.e., 10 or 12 items), we calculated the proportion of correct responses. For adult data collected before January 2019, scores were divided by 12; adult data collected after January 2019 were divided by 10. Higher proportions indicate better affective ToM ability. Missing responses were treated at the trial level as incorrect.

## Results

### Prediction 1: cognitive ToM would remain relatively stable from preschool to adulthood, with modest declines in older adulthood

**Figure 2:**
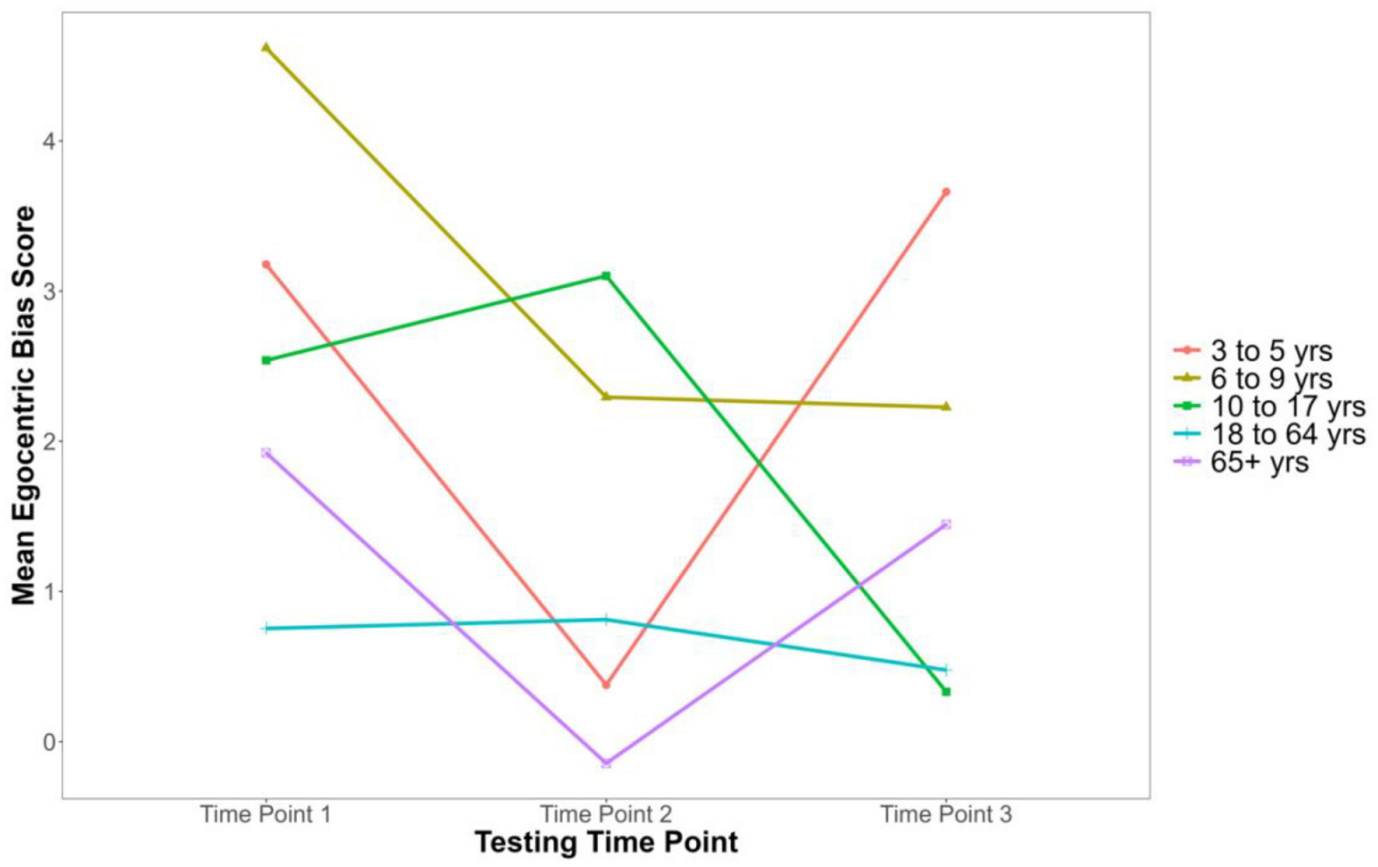
Mean egocentric bias scores in the Sandbox Task across time points for age groups. This graph is unscaled to emphasize differences between age groups and time points.

To compare differences in cognitive ToM ability across time points within different age groups, we conducted a 3 [Time Point: Time 1, Time 2, Time 3 (within)] × 5 [Age Group: 3–5 years, 6–9 years, 10–17 years, 18–64 years, 65+ years (between)] mixed-design ANOVA with egocentric bias as the dependent variable^2^. Assumptions of normality, homogeneity, and sphericity were violated; to avoid an inflated Type 1 error rate, a Greenhouse-Geisser correction was applied to adjust the degrees of freedom (Myers et al., 2010). There was a significant main effect of Age Group, *F*_(4,182)_ = 2.959, *p* = 0.021, η^2^G = 0.021 (see Table 1 for descriptive statistics). The main effect of Time Point was not significant, *F*_(1.62,295.29)_ = 1.932, *p* = 0.155, η^2^G = 0.007. Thus, egocentric bias scores did not significantly change across Time Points. Further, the interaction between Age Group and Time Point was not significant, *F*_(6.49,295.29)_ = 1.214, *p* = 0.297, η^2^G = 0.018 (see Figure 2 for visual representations of these trends). Thus, the pattern of differences in egocentric bias scores between Age Groups remained consistent across Time Points.

**Table 1:** Means and standard deviations summary statistics for egocentric bias scores in the Sandbox Task for age groups across time points.

**Age group**	***N***	**Time 1**	**Time 2**	**Time 3**
3–5 years	31	3.18 *(9.74)*	0.377 *(12.1)*	3.66 *(5.86)*
6–9 years	40	4.62 *(7.20)*	2.29 *(9.66)*	2.23 *(5.67)*
10–17 years	32	2.54 *(5.33)*	3.10 *(7.12)*	0.332 *(2.71)*
18–64 years	58	0.753 *(5.66)*	0.812 *(4.91)*	0.477 *(2.11)*
65+ years	26	1.92 *(5.60)*	−0.144 *(2.59)*	1.45 *(3.45)*

Data are presented as means; italicized values are standard deviations (SD).

Pairwise comparisons were conducted to investigate significant differences between Age Groups (see Table 2). A Bonferroni correction was applied to account for multiple comparisons (α = 0.004). After adjustment, only comparisons between the 6–9 years and 18–64 years groups were statistically significant, (*p* = 0.0021, *d* = 0.395) ^3^. Participants aged 6–9 years exhibited higher egocentric bias scores than those aged 18–64 years, suggesting poorer cognitive ToM abilities in the younger age group.

**Table 2:** Pairwise comparisons for egocentric bias scores in the Sandbox Task.

**Age groups comparison**	***N1***	***N2***	***p* (unadjusted)**	**Significance level**
3–5 vs. 6–9	31	40	0.375	ns
3–5 vs. 10–17	31	32	0.709	ns
3–5 vs. 18–64	31	58	0.11	ns
3–5 vs. 65+	31	26	0.487	ns
6–9 vs. 10–17	40	32	0.197	ns
6–9 vs. 18–64	40	58	**0.0061**	^***^
6–9 vs. 65+	40	26	0.116	ns
10–17 vs. 18–64	32	58	0.233	ns
10–17 vs. 65+	32	26	0.731	ns
18–64 vs. 65+	58	26	0.466	ns

N1 and N2 represent the collapsed sample sizes for the first and second age groups in each comparison. ns, Not significant. ^***^p < 0.004 (Bonferroni threshold). Results significant at the Bonferroni threshold are bolded.

Given assumption violations in the standard ANOVA, we conducted a trimmed ANOVA using 20% trimmed means to account for outliers and non-normality. The trimmed ANOVA indicated the main effect of Age Group was no longer significant, *F*_(4,46.5834)_ = 1.5016, *p* = 0.2171. These results suggest that the significant main effect of Age Group observed in the standard ANOVA may have been influenced by outliers. However, there was a significant main effect of Time Point, *F*_(2,54.9906)_ = 3.3467, *p* = 0.0425. Thus, there was a significant difference in egocentric bias scores across the three time points, suggesting that the participants' scores varied and did not remain stable throughout testing waves. The interaction between Age Group and Time Point remained non-significant, *F*_(8,52.6446)_ = 0.4186, *p* = 0.9047.

Overall, these results suggest that cognitive ToM abilities remained relatively stable within participants over the three time points, as evidenced by the lack of a significant interaction between Age Group and Time Point in both the standard ANOVA and the trimmed ANOVA.

### Prediction 2: affective ToM would remain relatively stable from preschool to adulthood, with modest declines in older adulthood emerging earlier compared to cognitive ToM

**Figure 3:**
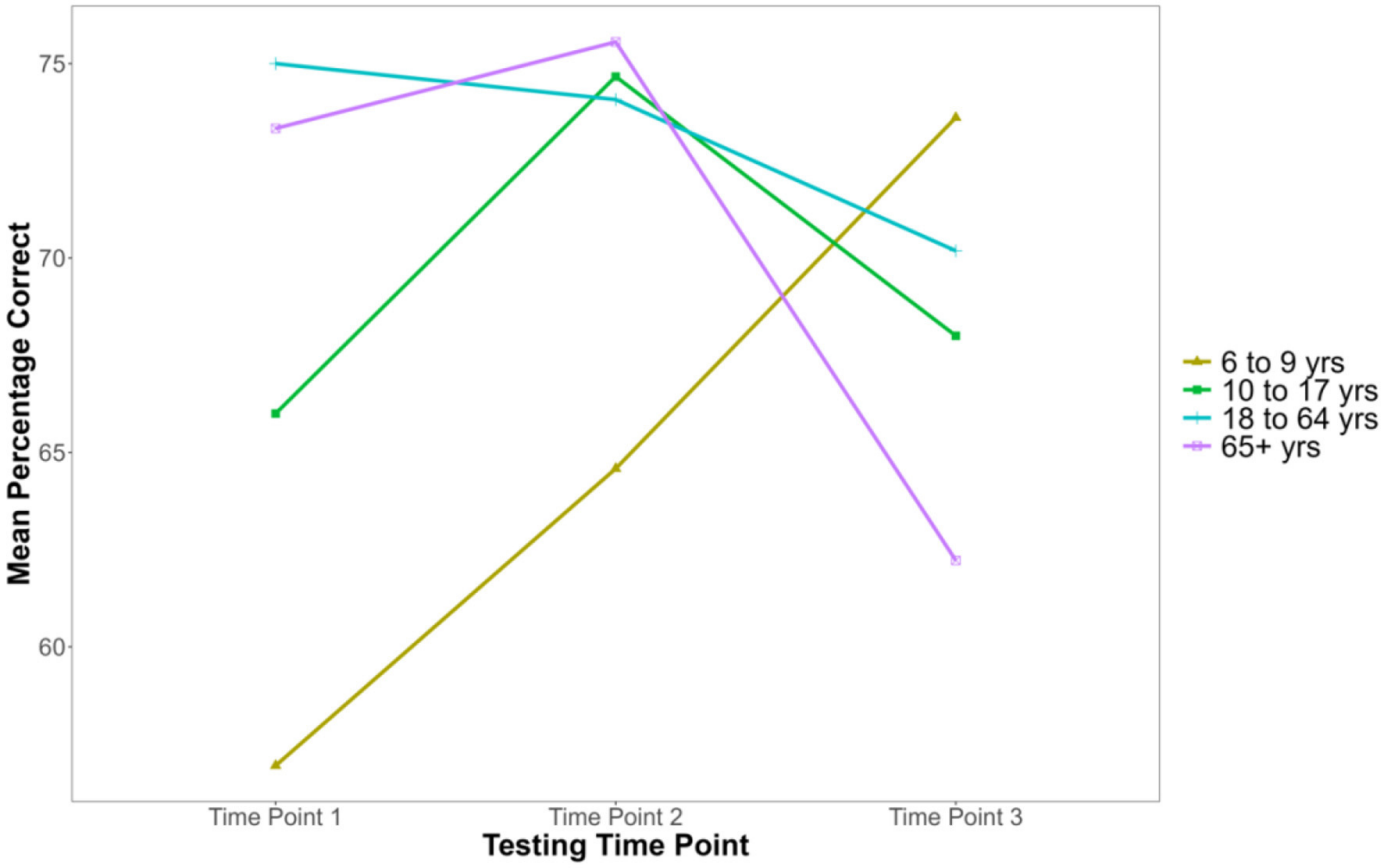
Mean percentage correct recognition response scores in the RMET across time points for age groups. This graph is unscaled to emphasize differences between age groups and time points.

To compare differences in affective ToM ability across time points within different age groups, we conducted a 3 [Time Point: Time 1, Time 2, Time 3 (within)] × 4 [Age Group: 6–9 years, 10–17 years, 18–64 years, 65+ years (between)] mixed-design ANOVA with the correct recognition response score on the Eyes task as the dependent variable. Assumptions of normality, homogeneity, and sphericity were met. There was a significant main effect of Age Group, *F*_(3,117)_ = 3.431, *p* = 0.019, η^2^G = 0.047. There was also a significant main effect of Time Point, *F*_(2,234)_ = 3.875, *p* = 0.022, η^2^G = 0.014. Thus, affective ToM differed significantly both between Age Groups and across Time Points (see Table 3 for descriptive statistics). Further, the interaction between Age Group and Time Point was significant, *F*_(6,234)_ = 8.112, *p* < 0.001, η^2^G = 0.083 (see Figure 3 for visual representations of these trends). Thus, the effect of Time Point on affective ToM varied across Age Groups.

**Table 3:** Means and standard deviations summary statistics for correct recognition response scores in the RMET for age groups across time points.

**Age group**	***N***	**Time 1**	**Time 2**	**Time 3**
6–9 years	36	56.9 *(14.0)*	64.6 *(15.2)*	73.6 *(12.5)*
10–17 years	25	66.0 *(13.4)*	74.7 *(15.1)*	*68.0 (14.8)*
18–64 years	45	75.0 *(16.9)*	74.1 *(15.6)*	70.2 *(16.1)*
65+ years	15	73.3 *(13.4)*	75.6 *(14.9)*	62.2 *(15.1)*

Data are presented as means; italicized values are standard deviations (SD).

Pairwise comparisons were conducted to investigate the interaction (see Table 4). A Bonferroni correction was applied to account for multiple comparisons (α = 0.004). After adjustment, statistically significant differences were observed at Time 1 between the 6–9 years and 18–64 years Age Groups, (*p* < 0.001, *d* = 1.154), and between the 6–9 years and 65+ years Age Groups, (*p* < 0.001, *d* = 1.186). These results indicate that participants aged 6–9 years exhibited lower correct recognition response scores compared to participants aged 18–64 and 65+ years, suggesting poorer affective ToM abilities in the younger group. No significant differences were found at Time 2 or 3.

**Table 4:** Pairwise comparisons for correct recognition response scores in the RMET across time points.

**Time point**	**Age groups comparison**	***N1***	***N2***	***p* (unadjusted)**	**Significance level**
1	6–9 vs. 10–17	36	25	0.0218	^*^
	6–9 vs. 18–64	36	45	**0.000000361**	^****^
	6–9 vs. 65+	36	15	**0.00053**	^****^
	10–17 vs. 18–64	25	45	0.0175	^*^
	10–17 vs. 65+	25	15	0.136	ns
	18–64 vs. 65+	45	15	0.709	ns
2	6–9 vs. 10–17	36	25	0.0128	^*^
	6–9 vs. 18–64	36	45	0.00649	^**^
	6–9 vs. 65+	36	15	0.0214	^*^
	10–17 vs. 18–64	25	45	0.877	ns
	10–17 vs. 65+	25	15	0.859	ns
	18–64 vs. 65+	45	15	0.746	ns
3	6–9 vs. 10–17	36	25	0.146	ns
	6–9 vs. 18–64	36	34	0.3	ns
	6–9 vs. 65+	36	15	0.0132	^*^
	10–17 vs. 18–64	25	45	0.553	ns
	10–17 vs. 65+	25	15	0.232	ns
	18–64 vs. 65+	45	15	0.0722	ns

N1 and N2 represent the sample sizes for the first and second age groups in each comparison. ns, Not significant. ^*^p < 0.05. ^**^p < 0.01. ^****^p < 0.001. Results significant at the Bonferroni threshold are bolded.

## Discussion

Using a longitudinal design, we explored the developmental patterns of cognitive and affective ToM across the lifespan. We extended previous work by employing continuous and consistent measures of ToM from preschool age through older adulthood. We predicted that ToM would remain relatively stable into adulthood, with modest declines in older adulthood. Our results partially supported these predictions, and revealed similarities in the developmental trajectories of cognitive and affective ToM.

As predicted, cognitive ToM remained relatively stable across the lifespan, as indicated by the non-significant interaction between Age Group and Time Point in the standard and trimmed ANOVAs. However, there was a significant main effect of Age Group in the standard ANOVA. Specifically, participants aged 6–9 years exhibited significantly lower cognitive ToM (higher egocentric bias scores) compared to the 18–64 years group. This suggests there are developmental improvements in cognitive ToM during childhood, followed by stability across adulthood. This finding largely aligns with developmental patterns reported in prior research using continuous measures. For example, Bernstein (2021) observed that cognitive ToM abilities (i.e., false-belief reasoning) remained relatively stable from preschool to older adulthood. Moreover, Bernstein et al. (2017) observed relative stability in ToM abilities across most of the lifespan, with modest declines emerging in older adulthood. Contrary to our prediction, there was no evidence of cognitive ToM decline in older adulthood. Pairwise comparisons revealed no significant differences in egocentric bias scores between younger adults (18–64 years) and older adults (65+ years). Thus, cognitive ToM appears to remain relatively stable across much of the lifespan past childhood, at least within the timeframe measured in this study using the Sandbox task. Notably, these results should be interpreted with caution given that the assumptions of the standard ANOVA were violated.

Similarly, our prediction for affective ToM was only partially supported, with its developmental patterns revealing similarities to cognitive ToM. Results revealed a significant interaction between Age Group and Time Point. Specifically, at Time 1, participants aged 6–9 years demonstrated significantly lower affective ToM (lower correct recognition responses) compared to the 18–64 and 65+ age groups. Notably, the significant difference for the 65+ age group was not found for cognitive ToM, demonstrating similar yet distinct developmental trajectories. However, no other pairwise comparisons were statistically significant at either time point, suggesting that, beyond childhood, affective ToM remains relatively stable. Indeed, contrary to our prediction, there was no evidence of affective ToM decline in older adulthood. Thus, neither ToM component demonstrated declines in older adulthood. Taken together, these results suggest that cognitive and affective components of ToM remain largely stable across the lifespan, with developmental changes occurring between childhood and adulthood.

Our findings align with prior research using continuous measures, which suggest that developmental patterns of ToM are relatively subtle (e.g., Bernstein et al., 2011; Dumontheil et al., 2010; Henry et al., 2013). This supports the view that continuous measures may better capture nuanced age-related changes compared to traditional discrete pass/fail measures. However, we acknowledge that this claim is premature, and that further work is needed to explore the differences between discrete and continuous measures of ToM. To address this, future studies could administer both continuous and discrete measures in a within-subject design to directly compare their ability to capture subtle changes in ToM across the lifespan. Using a wider variety of continuous (i.e., implicit) measures, such as reaction time (Kikuno et al., 2007), eye-tracking (Keysar et al., 2003), and mouse-tracking (van der Wel et al., 2014), would improve our understanding of nuanced age-related changes. Additionally, since continuous measures of ToM are less abundant, researchers might administer a battery of tasks that could then be combined into a continuous measure of ToM. This approach would also address the limitation of relying on a single discrete task. Overall, our findings emphasize the need to consider cognitive and affective ToM as distinct constructs that share similar developmental trajectories.

Beyond concerns related to measurement format (i.e., discrete vs. continuous), another important factor that may influence developmental patterns of ToM is task modality. Bottiroli et al. (2016) highlighted that differences in age-related ToM performance across studies may depend on whether the task relies on verbal or visual processing. Specifically, they proposed that abilities measured with verbal tasks (e.g., the Sandbox Task) remain relatively stable across the lifespan, as these tasks draw on verbal skills such as comprehension and social reasoning, which are relatively preserved with age. In contrast, performance on visual tasks (e.g., RMET) tends to decline earlier, as aging interferes with the ability to recognize emotions from facial expressions. Supporting this interpretation, Raimo et al. (2022) found that age-related declines in affective ToM were specific to tasks relying on visual modalities, whereas performance on verbal tasks remained relatively preserved. Future studies should carefully consider how task modality may affect observed developmental trajectories of ToM across the lifespan for both cognitive and affective ToM.

### Methodological considerations and limitations

We used a single measure each for cognitive and affective ToM across age groups to address a key concern in the ToM literature regarding whether previously reported developmental differences reflect true age-related changes. However, this approach is also a limitation because relying on only two measures cannot capture the full complexity of ToM. Including a wider range of tasks, such as the Strange Stories task (Happé, 1994) for cognitive ToM and the Movie for Assessment of Social Cognition (Dziobek et al., 2006) for affective ToM, would better capture different components of ToM in real-world social situations. Replicating our study with these diverse measures could also provide insights into other related ToM skills, such as hidden emotion and sarcasm, that were not observed here. That said, most tasks in the literature are not appropriate to measure ToM from young childhood to old age. We encourage future researchers to incorporate additional measures of ToM to replicate and extend our findings. We also urge researchers to develop more tasks that can measure ToM in preschoolers through older adults.

Another limitation of the present study was the use of the RMET to measure affective ToM. Psychometric research has raised concerns about the task's latent structure, failing to identify a well-fitting unidimensional or multidimensional factor structure (Higgins et al., 2023). Internal consistency, typically measured using Cronbach's alpha, has also varied widely across studies (Kittel et al., 2022). Furthermore, the RMET has limited sensitivity for discriminating among individuals with average to high levels of ToM ability; thus, it may not be an appropriate measure for non-clinical samples (Black, 2019). Relatedly, Oakley et al. (2016) showed that alexithymia (an impairment of facial recognition that co-occurs in autism spectrum disorder) accounts for differences between autism spectrum disorder and control subjects on the RMET. The authors suggest that the RMET assesses emotion recognition rather than ToM ability. Additionally, a systematic review highlighted that many studies using the RMET lack sufficient evidence of construct validity, raising concerns about the reliability of existing findings (Higgins et al., 2024). While we acknowledge these possibilities, we chose to include the RMET because it is widely used in the existing literature as a measure of affective ToM. Indeed, Baron-Cohen et al. (2001) argue that a relevant social context must be referenced from memory to understand the emotion. Further, populations with ToM deficits who score lower on the RMET compared to typically developing controls have shown comparable scores on measures of basic emotion labeling and gender-recognition control tasks (Baron-Cohen et al., 2001, 1997a,b). Ultimately, the question of whether the RMET measures ToM or emotion recognition is an important one but is beyond the scope of this work.

Finally, we acknowledge that some of our age group sample sizes were small, particularly for older adults. Thus, we had limited power to detect subtle age-related differences. Using G^*^Power analysis (Faul et al., 2007), we conducted sensitivity analyses for each pairwise comparison between the 65+ group and other age groups. These analyses revealed that statistical power was low across all such comparisons, indicating that our study was not sufficiently powered to detect small or even medium-sized effects involving older adults. As such, the findings related to this group should be interpreted with caution. Nonetheless, given the scarcity of longitudinal research on ToM in adults, our findings provide a valuable foundation for future studies to build from.

## Conclusion

This study explored the developmental trajectory of ToM across the lifespan to explore age-related changes in ToM ability, both within and across age groups. We included separate measures of cognitive and affective ToM and used the same tasks across different age groups, spanning preschool to older adulthood. Our findings suggest that both cognitive and affective ToM remain relatively stable across the child to older adult lifespan. For both ToM components, the most pronounced developmental changes occurred during childhood, with younger children showing poorer ToM abilities compared to adults.

While this study addresses gaps in the ToM literature by using consistent measures across a diverse set of age groups, limitations, such as the reliance on a limited set of tasks and concerns about task validity, highlight the need for further research. Our results need to be replicated using more diverse methodologies. Nevertheless, our results add to a growing body of literature showing similar, yet distinct developmental trajectories for cognitive and affective ToM. Moreover, our work highlights the value of continuous ToM measures in capturing subtle changes across the lifespan.

## Data Availability

The raw data supporting the conclusions of this article will be made available by the authors, without undue reservation.

## Footnotes

^1^This pattern varies slightly across cultures. Individuals from collectivist cultures are more likely to understand knowledge access before diverse beliefs, compared to those from individualist cultures (Shahaeian et al., 2011; Wellman et al., 2006).

^2^Analyses controlling for delays between time points and the number of questions asked are presented in the Supplementary materials.

^3^See Table 2 for all pairwise comparisons, including those significant before Bonferroni adjustment.
