# Supplementary material for: A longitudinal study of theory of mind across the lifespan
Source: Front Psychol. 2025 Jul 17;16:1549378. doi: 10.3389/fpsyg.2025.1549378 (PMC12312641; doi:10.3389/fpsyg.2025.1549378)

Supplementary Material

1 Supplementary Data

Sandbox Task

a. **Primary analysis with additional participants**
In our updated sample, we had $N = 194$ participants (30 *3 to 5 year olds*, 43 *6 to 9 year olds*, 35 *10 to 17 year olds*, 60 *18 to 64 year olds*, and 26 *65+ year olds*. Re-running our primary analysis with this larger sample (particularly in the oldest age group) revealed a main effect of Age Group, $F(4, 189) = 2.46$, $p = .047$, such that *6 to 9 year olds* exhibited higher egocentric bias than *18 to 64 year olds.* There was no significant main effect of Time, $F(2, 380) = 2.42$, $p = .09$, and no significant interaction between Age Group and Time, $F(8, 379) = .74$, $p = .65$. These results closely mirror those of our main analysis.

b. **Delays between time points**
We also examined the delays between time points for different age groups in our updated sample (see SM Figure 1). The average delay between testing waves was 2.17 years ($SD = 1.73$). Comparing the Time 1-2 and Time 2-3 delays by age group, there was a significant main effect of age group, $F(4, 185) = 5.69$, $p < .001$, such that *3 to 5 year olds* had a longer average delay between time points than *10 to 17 year olds*, and all age groups older than 5 years had a shorter average delay between time points than *65+ year olds*. There was also a significant main effect of time, $F(1, 187) = 13.05$, $p < .001$, such that on average, the delay between Time 1-2 was shorter than the delay between Time 2-3. There was no interaction between Age Group and Time, $F(4, 186) = .21$, $p = .93$.

c. **Controlling for testing delays, and whether participants were asked 1 vs. 2 questions**
Of the 583 unique testing instances in the updated data set, 223 (38%) had two questions per trial. In a model predicting egocentric bias from time and age group, controlling for both number of questions and delays between time points (setting delay to 0 for Time Point 1), the main effect of Age Group was no longer significant, $F(4, 179) = 2.16$, $p = .08$. Additionally, when controlling for these variables, the main effect of Time Point became significant, $F(2, 485) = 9.62$, $p < .001$ (although no individual pairwise comparisons achieved significance when controlling for familywise error rate). Additionally, there was a significant interaction between Time Point and delay, $F(1, 553) = 3.93$, $p = .048$. Follow-up tests on this interaction revealed that the effect of delay was significant at Time Point 2, $F(1, 193) = 12.72$, $p < .001$, such that longer delays since Time Point 1 were associated with greater egocentric bias, but that the effect of delay was *not* significant at Time Point 3, $F(1,$

192) = .81, $p = .37$. There were no other significant main effects or interactions, $Fs < 2.87$, $ps > .07$. Despite the delay effects, results overall resembled those reported for our main analysis, except the pattern for 65+ year olds (perhaps due to the low n in this age group; please see SM Figure 2).

Reading the Mind in the Eyes Task (RMET)

a. Primary analysis with additional participants

In our updated sample, we had $N = 133$ participants (41 *6 to 9 year olds*, 25 *10 to 17 year olds*, 48 *18 to 64 year olds*, and 19 *65+ year olds*. Re-running our primary analysis with this larger revealed a main effect of Age Group, $F(3, 129) = 6.17$, $p < .001$, such that *6 to 9 year olds* exhibited lower RMET scores than *18 to 64 year olds.* The main effect of Time was no longer significant, $F(2, 258) = .89$, $p = .41$, but the interaction between Age Group and Time remained significant, $F(6, 258) = 4.16$, $p < .001$. Follow-up pairwise tests revealed that at Time 1, *6 to 9 year olds* showed significantly lower RMET scores than *18+ year olds* ($ps < .04$), and at Time 2, *6 to 9 year olds* showed significantly lower RMET scores than *18 to 64 year olds* ($p = .01$). These results closely mirror those of our main analysis.

b. Delays between waves

The average delay between testing waves was 2.3 years ($SD = 1.67$; see SM Figure 3). Comparing the Time 1-2 and Time 2-3 delays by age group, there was a significant main effect of age group, $F(3, 125) = 3.65$, $p =.01$, such that *65+ year olds* had a longer average delay than *6 to 9 year olds*. There was also a significant main effect of time, $F(1, 125) = 62.14$, $p < .001$, such that on average, the delay between Time 1-2 was shorter than the delay between Time 2-3. There was also an interaction between age group and Time, $F(3, 125) = 4.37$, $p = .006$, such that the difference between Time 1-2 and Time 2-3 delays was larger for *65+ year olds* than *6 to 9 year olds* and *10 to 17 year olds*.

c. Controlling for testing delays

Of the 399 unique testing instances in our updated sample, only 1 used the 10-question version of the RMET, so we did not conduct any analyses controlling for questions. In an analysis controlling for testing delays, we observed main effects of age group, $F(3, 127) = 7.53$, $p < .001$, and time, $F(2, 277) = 3.43$, $p = .03$, consistent with our primary analyses. However, the interaction between age group and time was no longer significant, $F(6, 280) = 1.03$, $p = .40$. Additionally, we observed a significant main effect of delay, $F(1, 352) = 10.23$, $p = .002$, such that longer delays between waves was associated with lower RMET scores. However, given that our *65+ year old* participants had the lowest RMET scores at Time 3 *and* the longest delays between testing, it is possible that this effect is the result of confounded age and delays. Indeed, there was a significant age group X delay interaction, $F(3, 352) = 3.40$, $p = .02$, such that increased delays led to significantly *higher* RMET scores for *6 to 9 year olds* ($p = .03$) but significantly *lower* RMET scores for *18 to 64 year olds* ($p <$

.001) and *65+ year olds* ($p < .001$). These results suggest that additional (longer) development benefitted children but not adults and older adults. But qualitatively, results were very similar to those of our primary analysis (see SM Figure 4).

2 Supplementary Figures and Tables

SM Figure 1. Delay Between Time Points by Age Group in the Sandbox Task.

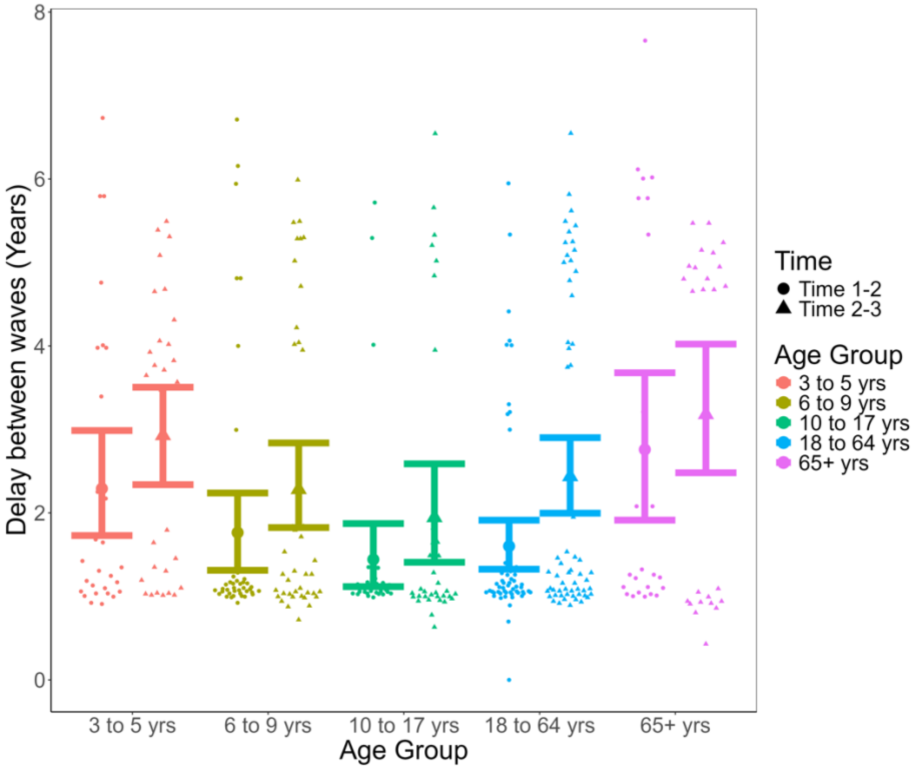

SM Figure 2. Mean egocentric bias scores in the Sandbox Task across Time Points for Age Groups, controlling for testing delays and number of questions.

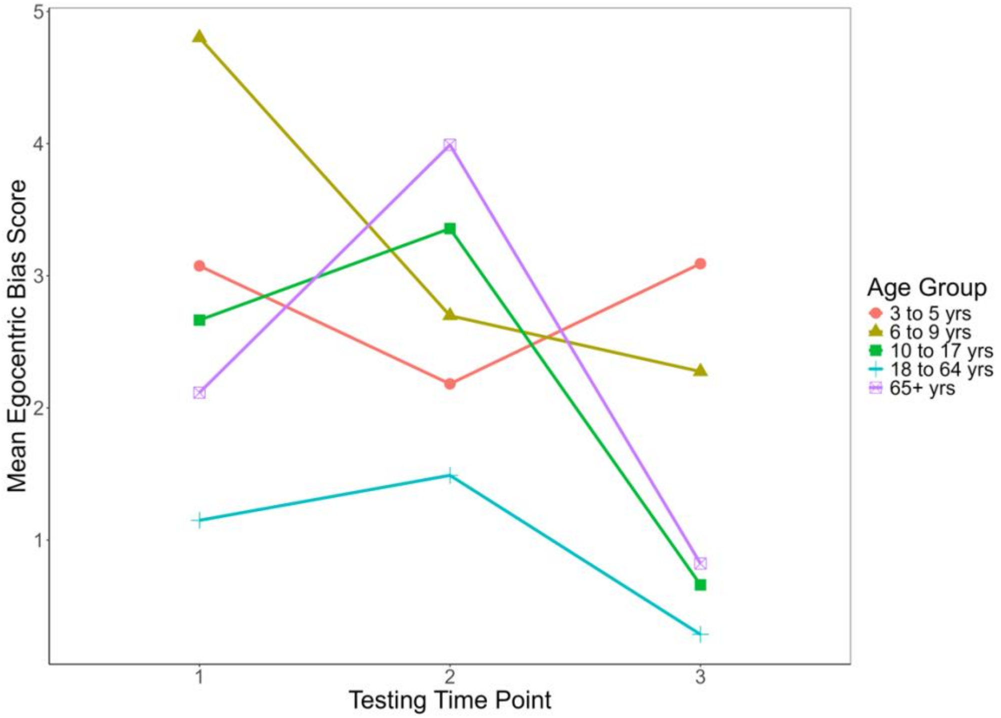

SM Figure 3. Delay Between Time Points by Age Group in the RMET.

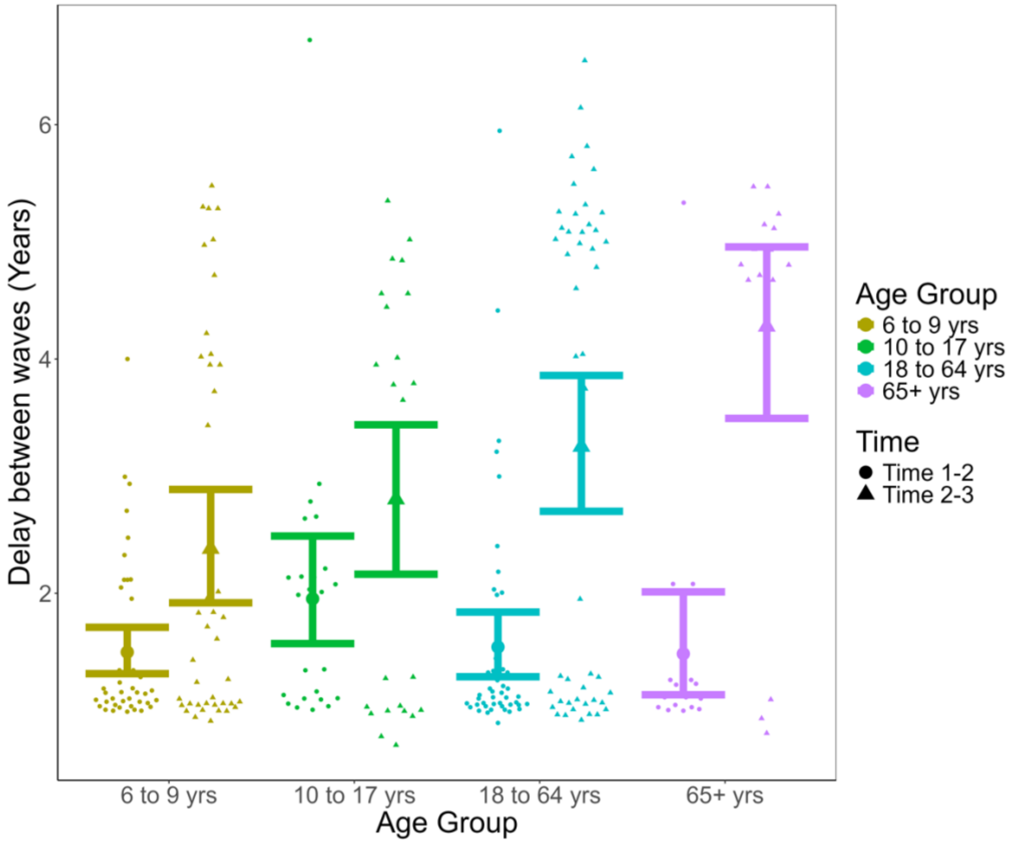

SM Figure 4. Mean percentage correct recognition response scores in the RMET across Time Points for Age Groups, controlling for testing delays and number of questions.

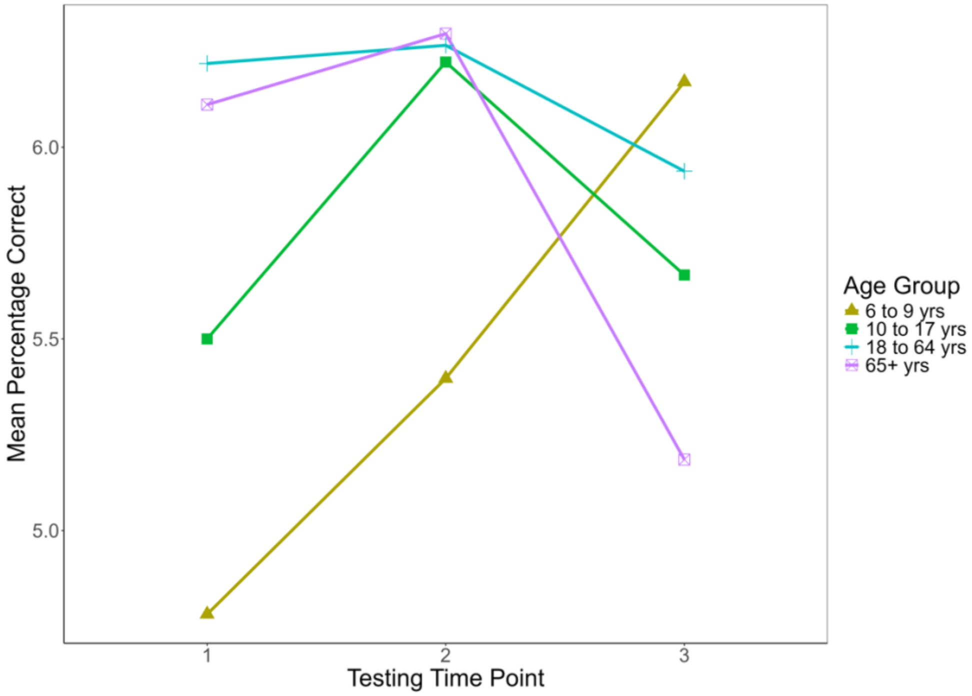

Supplement: Supplementary file 1 [file Data_Sheet_1.pdf]
